# Editorial: Infectious diseases and cancer: convergence and divergence between bacteria, viruses and helminths

**DOI:** 10.3389/fonc.2024.1471156

**Published:** 2024-08-29

**Authors:** Ana Elena Escorcia-Saucedo, Alberto N. Peón, Aldo Christiaan Jardínez-Vera, Luis I. Terrazas, José L. Medina-Franco

**Affiliations:** ^1^ Sociedad Española de Beneficencia, Pachuca, Hidalgo, Mexico; ^2^ Hospital Español de Pachuca, Pachuca, Hidalgo, Mexico; ^3^ Área Académica de Medicina, Universidad Autónoma del Estado de Hidalgo, Pachuca, Mexico; ^4^ Laboratorio de Modelado y Simulación Computacional en Nanomedicina, Escuela Superior de Apan, Licenciatura en Ingeniería en Nanotecnología, Universidad Autónoma del Estado de Hidalgo, Apan, Hidalgo, Mexico; ^5^ Unidad de Biomedicina, Facultad de Estudios Superiores Iztacala, Universidad Nacional Autónoma de México, Tlalnepantla, Estado de México, Mexico; ^6^ Departamento de Farmacia, Facultad de Química, Universidad Nacional Autónoma de México, México, Mexico

**Keywords:** cancer, immunity, helmints, virus, immune regulation

## 1 Introduction

Interestingly, while cancer may develop in complete independence to other diseases, some infectious diseases have the potential to increase the susceptibility to cancer. For instance, the human papillomavirus (HPV) has been linked to several types of cancer (1–6), as well as some other parasite types like the bacteria Helicobacter pylori (7–9), or parasitic worms like *Schistosoma* spp., *Clonorchis sinensis* and *Opistorchis viverrini*. Or even the fungi *Chlamydia trachomatis* (10), both as an independent risk factor and as a risk enhancer for the HPV infections. Importantly, as research progresses, more pathogens will be added detected to corelate with cancer development.

Moreover, 15.4% of the new cancer cases in 2012 were related to infections (11), which based on GLOBOCAN statistics is equivalent to an estimated of 2.2 million cases each year; and it is estimated that by 2050 most of the cancers will be produced by infections (12). The most studied microorganisms by the aforementioned organization are pathogens that have been classified as Group 1 human carcinogens, among of which ≈810,000 cases were related to *Helicobacter pylori*, ≈690,000 cases to HPV and ≈210,000 cases to “other agents” which includes *Schistosoma spp*, *O. viverrini*, and *C. sinensis* (13).

On the other hand, the hallmarks of cancer are conceptual frameworks that organize the understanding about the neoplastic disease in a multistep process. Since the year 2000, when Hanahan & Weinberg (14) made the first proposal that included only six hallmarks, four more have been added (15). Currently sustaining proliferative signaling, evading growth suppressors, avoiding immune destruction, tumor-promoting inflammation, resisting cell death, enabling replicative immortality, inducing angiogenesis, activating invasion and metastasis, enhanced genome instability/mutations, and deregulating cellular energetics are recognized as steps in the cellular transformation process that have to be undertaken in order for a tumor to develop.

Interestingly, many inflammatory mediators possess the ability to induce some of the events that have been described as hallmarks of cancer. Thus, some pathogens may be able to stimulate malignancy induction and metastasis independently of their ability to produce cancer-inducing virulence factors, but in dependance on the immune response that they evoke. Such phenomenon is recognized as tumor-promoting inflammation and may be as diverse as the wide variety of the parasites that have been linked to cancer.

In this way, it may be important to understand the fine tuning of the host-parasite microenvironment in order to develop treatments and preventive measures for infection-related cancers, taking into account that not every infectious agent produces virulence factors related to cancer development, but all of them elicit an immune response, and such set of phenomena may have a causal relationship with cancer.

## 2 Contents of the Research Topic

In this Research Topic we had the privilege to publish two excellent original research articles, as well as a very detailed review and an interesting hypothesis article. And all these works exemplarily sum to the current knowledge on the field of the host-parasite immunological interface-related cancer development.

For instance, Han et al. show that genes related to milder COVID-19 forms do correlate with a reduced risk to develop head and neck cancer. Interestingly, such genes associate with a strong immune response to viral infections, mainly through the response to type 1 interferons, which have been shown to play a critical role in the host’s defense against SARS-CoV-2 (16). These findings exemplify the fact that severe and/or persistent viral infections may share pathways with cancer development, paving the way for the successive articles in this Research Topic.

At the other end of the spectrum, we hereby present an interesting original investigation made by Aragón-Franco et al., where they studied the *Toxocara canis*-elicited changes on the immune response and their relationship with cancer. They found that this parasite’s secreted/excreted products are able to promote tumor vascularization in relation to an enhanced production of vascular endothelial growth factor. Such increased vascularization was later found to be associated to metastasis in the lungs.

On a similar line of work, Esperante et al. in an elegantly written review, highlight many of the similarities and divergences between the immuno-metabolic profile of helminth-derived infestations and cancer immunity, pinpointing at the fact that both induce Th2-type immune responses, as well as enhanced glucose deprivation, fatty acid oxidation and oxidative phosphorylation. And such changes converge on the induction of an impaired immune response that is not able to fully eliminate these agents.

Finally, Jiang and Wu contributed to the Research Topic with an interesting hypothesis article, where they elaborate on an old hypothesis of the German pathologist Otto Aichel, in the light of newer findings. Such hypothesis explains that cancer cells may acquire their metastatic ability by fusing with leukocytes. In their research, Jiang and Wu found a plausible explanation for this hypothesis, where immunologically killed cancer cells may be phagocytosed by memory macrophages which, because of their mainly tolerogenic phenotype, may not fully degrade the cancer cell’s debris, leading to the establishment of a tetraploid cell with migrating capabilities.

In such a context, we can envisage a panorama where the properties of the immunological response to cancer are deeply altered by parasites of either viral or helminthic types, favoring not only malignancy induction, but also metastasis. In none of the research products presented hereby a specific mutagenic substance was identified, but many immune-regulatory mechanisms that foster cancer development were identified.

More research is needed to find common immunological mechanisms behind infection-related cancer induction, but the identification of such pathways may lead to the development of a new generation of immunological enhancing drugs to fight cancer.
